# Imbalance of dendritic cell co-stimulation in COPD

**DOI:** 10.1186/s12931-015-0174-x

**Published:** 2015-02-07

**Authors:** Paul Stoll, Martin Ulrich, Kai Bratke, Katharina Garbe, J Christian Virchow, Marek Lommatzsch

**Affiliations:** Abteilung für Pneumologie und Internistische Intensivmedizin, Zentrum für Innere Medizin, Universität Rostock, Ernst-Heydemann-Str. 6, 18057 Rostock, Germany

**Keywords:** COPD, Dendritic cells, Chronic inflammation, Emphysema

## Abstract

**Background:**

Dendritic cells (DCs) control immunity and play a role in the pathogenesis of chronic obstructive pulmonary disease (COPD). However, the expression of function-associated surface molecules on circulating DCs in COPD is unknown.

**Methods:**

Four-colour flow cytometry was used to compare blood DC surface molecules of 54 patients with COPD (median age: 59 years; median FEV_1_: 38% predicted, median CAT score: 24) with two age-matched control groups with normal lung function: 21 current smokers and 21 never-smokers.

**Results:**

Concentrations of plasmacytoid DCs (pDCs) and myeloid DCs (mDCs) and the mDC/pDC ratio did not differ between the groups. The increased expression of BDCA-1, BDCA-3, CD86 and CCR5 on mDCs in patients with COPD did not significantly differ from smokers with normal lung function. In contrast, COPD was specifically characterised by a decreased expression of the anti-inflammatory co-stimulatory molecule PD-L1 on pDCs and an increased expression of the pro-inflammatory co-stimulatory molecule OX40 ligand (OX40L) on mDCs. These changes were not confined to patients with elevated systemic inflammation markers (leukocytes, c-reactive protein, interleukin-6, fibrinogen). The ratio of OX40L to PD-L1 expression (OX40L/PD-L1 ratio), a quantitative measure of imbalanced DC co-stimulation, correlated with the severity of pulmonary emphysema in patients with COPD.

**Conclusion:**

An imbalance of DC co-stimulation might contribute to the pathogenesis of COPD.

**Electronic supplementary material:**

The online version of this article (doi:10.1186/s12931-015-0174-x) contains supplementary material, which is available to authorized users.

## Introduction

Chronic obstructive pulmonary disease (COPD) is a chronic inflammatory disorder characterised by both local abnormalites (including a specific type of airway inflammation) and systemic abnormalities (including typical co-morbidities and various degrees of systemic inflammation) [1]. An abnormal immune response to inhaled noxious agents is a central pathogenetic feature of COPD, leading to lymphoid follicle formation around the airways and small airway obstruction [1]. Smoking is the most important risk factor for the development of COPD. However, less than 30% of all smokers develop COPD [2] and it is still unclear which specific immunologic features account for COPD development.

Dendritic cells (DCs), which can be subdivided into myeloid DCs (mDCs) and plasmacytoid DCs (pDCs), control pulmonary immune responses. DC accumulation and activation in the airways has been postulated to be a driver of COPD immunopathology [3-9]. Using an established method to analyse human bronchoalveolar lavage fluid DCs [10-13], we have shown that airway mDCs of asymptomatic smokers show a profound adaptation to cigarette smoke exposure, including a strong upregulation of antigen-recognition receptors such as CD1a, BDCA-1 or Langerin and co-stimulatory molecules such as CD86 [11]. Of note, this upregulation was also found on airway mDCs of current smokers with COPD, but not former smokers with COPD, suggesting that these changes are related to smoke exposure, rather than the presence of COPD. In addition, this upregulation correlated with less airway obstruction, further supporting the concept that an upregulation of these markers is not related to COPD pathogenesis [14]. Thus, the specific causes of chronic airway inflammation in COPD are still enigmatic. It has been postulated that enhanced local concentrations of chemokines contribute to the accumulation of DCs and the subsequent activation of lymphoyctes [3]. We hypothesised that there may be additional changes of co-stimulatory molecules on circulating DCs contributing to the immune dysregulation in COPD. It was the aim of the present study, therefore, to study major co-stimulatory molecules such as programmed death ligand 1 (PD-L1) or OX40 ligand (OX40L) and other function-associated surface molecules on circulating DCs of patients with COPD for the first time, and to compare the findings with control groups of asymptomatic smokers and never-smokers.

## Methods

### Participants

Controls were recruited using public notices in Rostock (Germany). Patients with stable COPD were recruited at the University Hospital of Rostock (Germany). Inclusion criteria for patients with COPD were as follows: 1. doctor’s diagnosis of COPD, 2. age between 40 and 85 years, 3. smoking history of at least 5 pack years, 3. a FEV_1_/FVC ratio < 70% after inhalation of a short-acting beta-agonist. Former smokers were defined as subjects who quit smoking at least 4 weeks before study inclusion. Exclusion criteria for patients with COPD were: 1. presence of any malignant diseases, 2. any signs of an infection/exacerbation or any treatment with antibiotics or short-term corticosteroids within the last 4 weeks prior to enrollment. Controls were recruited using the following inclusion criteria: 1. no history of chronic respiratory diseases, 2. age between 40 and 85 years, 3. no history of smoking and no exposure to smoking partners or relatives at home (never-smoking controls) or a smoking history of at least 5 pack years and current smoking of at least 15 cigarettes per day (asymptomatic current smokers). For both control groups, exclusion criteria were as follows: 1. presence of any malignant or chronic inflammatory diseases, 2. any signs of an infection or treatment with antibiotics within the last 4 weeks prior to enrollment. The study was approved by the local ethics committee of the Ärztekammer Mecklenburg-Vorpommern, Rostock (Germany). All participants gave their written informed consent.

### Study design

All participants were examined between 8 am and 12 pm. Lung function tests and blood sampling were performed on the same day. In a first step, informed consent was obtained and a structured history was taken. Subsequently, body plethysmography was performed and the diffusion capacity (DLCO) measured (Masterscreen, Jaeger, Carefusion, Hoechberg, Germany). Then, peripheral blood was taken for laboratory analyses (differential blood counts, fibrinogen, c-reactive protein, Interleukin-6 and 25-OH-Vitamin D) and for the analysis of blood DCs and T cells.

### Flow cytometry

Blood dendritic cells were analysed using freshly collected EDTA blood as described [10-13], using the antibodies detailed in the Additional file 1: Table S1. Among blood cells negative/dim for lineage markers (CD3, CD14, CD16, CD19, CD20, CD56), mDCs were defined as CD11c^+^HLA-DR^+^lin^neg/dim^ cells and pDCs as CD123^+^HLA-DR^+^lin^neg/dim^ cells (Additional file 1: Figure S1). Surface molecule expression on DCs was quantified in histogram plots using isotype control antibodies to discriminate between specific and non-specific staining (Additional file 1: Figures S2 and S3). CD4-positive T cells in peripheral blood were defined as CD3^+^CD4^+^ cells, regulatory T cells (Tregs) were defined as CD3^+^CD4^+^CD25^++^CD127^low/neg^ cells (Additional file 1: Table S1).

### Statistical analysis

Statistical analysis was performed using SPSS Statistics (SPSS Inc., Chicago, Illinois, USA). The majority of parameters was not normally distributed. Therefore, parameters were expressed as medians (minimum – maximum). For the comparison of groups, the Mann–Whitney-U test for unrelated samples was used. Correlation analyses were performed using Spearman’s correlation coefficient. Probability values of p < 0.05 were regarded as significant. Boxplots display the median (line within the box), interquartil range (edges of the box) and extremes (vertical lines). Significant differences between the groups are marked with the p-value.

## Results

### Characteristics of the participants

Twenty-one never-smokers, 21 asymptomatic current smokers and 54 patients with the diagnosis of COPD were included in the study. Characteristics of the participants are detailed in Table 1. There were no differences in age and gender distribution between the groups. There was no difference in the number of pack years (PY) between the group of asymptomatic smokers and the group of patients with COPD. Smokers displayed a higher body mass index (BMI) than never-smokers and patients with COPD. Patients with COPD displayed significantly worse lung function parameters, as compared to both control groups, with a median FEV_1_ of 1,0 liters (38% predicted): 14 patients were in spirometric GOLD stage II, 26 patients in spirometric GOLD stage III und 14 patients in spirometric GOLD stage IV. The median CAT (COPD Assessment Test) score in the COPD group was 24 (Table 1). One patient was treated with a long-acting beta-agonist (LABA) monotherapy, 3 patients with a long-acting muscarinergic antagonist (LAMA) monotherapy, 18 patients with a LABA/LAMA combination therapy, one with a LABA/ICS combination therapy and 30 patients with a LABA/LAMA/ICS triple therapy. Ten patients (18.5%) were treated with oral corticosteroids (OCS) and 31 patients (57.4%) with ICS (Table 1).

**Table 1:** **Characteristics of the participants**

	**Never-smoker (N)**	**Smoker (S)**	**COPD (C)**	**S vs. N**	**C vs. N**	**C vs. S**
	**n = 21**	**n = 21**	**n = 54**	**p-value**	**p-value**	**p-value**
**Age** (years)	63 [50–79]	61 [51–75]	59 [40–85]	0.337	0.274	0.859
**Gender** (male/female)	12/9	13/8	33/21	0.753	0.753	0.949
**BMI** (kg/m^2^)	26 [21–32]	29 [18–39]	25 [15–41]	<0.05	0.683	<0.05
**Waist circumference** (cm)	90 [66–125]	101 [72–115]	91 [64–130]	<0.05	0.432	0.102
**Pack-years** (PY)	0 [0–0]	39 [23–69]	38 [5–80]	<0.001	<0.001	0.262
**Smoking status** (NS/CS)	0 / 21	21 / 0	12 / 42	<0.001	<0.05	<0.001
**LAMA/LABA/ICS **(No. treated)	0/0/0	0/0/0	51/50/31	NA	<0.001*	<0.001*
**THEO/ROF/OCS** (No. treated)	0/0/0	0/0/0	12/8/10	NA	<0.05**	<0.05**
**mMRC**	0 [0–1]	0 [0–2]	2 [1–4]	0.076	<0.001	<0.001
**CAT**	4 [0–14]	7 [1–20]	24 [7–35]	0.050	<0.001	<0.001
**FEV**_**1**_ (Liters)	3.2 [1.9-5.1]	2.9 [1.8-3.9]	1.0 [0.5-2.4]	0.113	<0.001	<0.001
**FEV**_**1**_ (% predicted)	112 [84–133]	98 [66–124]	38 [16–75]	<0.01	<0.001	<0.001
**FEV**_**1**_ **%FVC** (%)	81 [73–86]	80 [71–94]	49 [28–69]	0.521	<0.001	<0.001
**MEF**_**50**_ (% predicted)	81 [45–148]	75 [29–123]	12 [5–33]	0.122	<0.001	<0.001
**RV** (% predicted)	99 [29–145]	107 [64–141]	199 [76–347]	0.119	<0.001	<0.001
**TLC** (% predicted)	103 [71–120]	98 [68–127]	118 [79–164]	0.571	<0.001	<0.001

Parameters are displayed as median values [minimum…maximum]. *Abbreviations denote:* Long-acting muscarinergic antagonist (LAMA), Long-acting Beta-agonist (LABA), Inhaled corticosteroid (ICS), Theophyllin (THEO), Roflumilast (ROF), Oral corticosteroid (OCS), modified Medical research council (mMRC), COPD assessment test (CAT), Inspiratory vital capacity (IVC), Forced expiratory volume in the first one second (FEV1), ratio of the FEV1 to the forced vital capacity (FEV1/FVC), maximum expiratory flow at 50% of VC (MEF50), residual volume (RV), total lung capacity (TLC), Non-Smoker (NS), Current Smoker (CS). The columns on the right side of the table show comparisons between the groups: Asymptomatic smokers (S), never-smokers (N), patients with COPD (C). *p < 0.001 for the differences in medication with LAMA, LABA and ICS. **p < 0.05 for differences in medication with OCS and Theophyllin.

### Markers of systemic inflammation

Blood concentrations of leukocytes, C-reactive protein (CRP) and fibrinogen were elevated compared to never-smoking controls, but did not significantly differ between asymptomatic smokers and patients with COPD (Table 2 and Additional file 1: Figure S4). In addition, there were no significant differences in the percentages of neutrophils, eosinophils and lymphocytes among blood leukocytes between asymptomatic smokers and patients with COPD (data not shown). In contrast, blood concentrations of Interleukin-6 (IL-6) were significantly higher in patients with COPD, as compared with asymptomatic smokers and never-smokers (Table 2 and Additional file 1: Figure S4). Twenty-seven patients with COPD (50%), 9 asymptomatic smokers (43%) and 1 never-smoker (5%) displayed a systemic inflammation according to the definition proposed by Agusti and colleagues [15] (at least two elevated parameters: leukocytes > 8.6 10^6^/ml, c-reactive protein > 8.7 mg/l, fibrinogen > 5.18 g/l, Interleukin-6 > 2.6 pg/ml) (Table 2). Blood IL-6 concentrations (but not other markers of systemic inflammation) correlated with lung function parameters (FEV_1_ % predicted: r = −0.33, p = 0.015; IVC % predicted: r = −0.42, p = 0.002; MEF_50_ % predicted: r = −0.27, p = 0.049), the 6 minute walk test (6-MWT) distance (r = −0.51, p = 0.001), the CAT score (r = 0.34, p = 0.012) and the mMRC score (r = 0.41, p = 0.002) in patients with COPD (Additional file 1: Figure S5). There was no significant difference in the total concentration of Tregs and in the percentage of Tregs among CD4-positive T-cells in peripheral blood between the 3 groups (Additional file 1: Figure S6).

**Table 2:** **Blood parameters**

	**Never-smoker (N)**	**Smoker (S)**	**COPD (C)**	**S vs. N**	**C vs. N**	**C vs. S**
	**n = 21**	**n = 21**	**n = 54**	**p-value**	**p-value**	**p-value**
**Systemic inflammation** (yes/no)	1/20	9/12	27/27	<0.01	<0.001	0.530
**Leukocytes** (10^9^/l blood)	6.1 [4.0-9.1]	8.7 [4.4-13.7]	8.4 [4.1-13.9]	<0.01	<0.001	0.567
**CRP** (mg/l blood)	1.0 [1.0-8.2]	3.7 [1.0-14.8]	3.5 [1.0-24.3]	<0.001	<0.001	0.552
**Fibrinogen** (g/l blood)	3.0 [2.4-4.8]	3.6 [2.5-5.4]	4.2 [2.2-6.4]	<0.05	<0.001	0.153
**Interleukin-6** (pg/ml blood)	2.17 [1.50-5.62]	2.42 [1.50-6.94]	4.19 [1.5-14.4]	0.362	<0.001	<0.01
**25-OH-Vitamin-D** (nmol/l)	54 [24–112]	62 [27–108]	45 [13–115]	0.443	0.294	0.059

Parameters are displayed as median values [minimum…maximum]. *Abbreviations denote:* C-reactive protein (CRP). Presence of systemic inflammation was defined according to the proposal by Agusti and colleagues [15]. The columns on the right side of the table show comparisons between the groups: Asymptomatic Smokers (S), Never-smokers (N), patients with COPD (C).

### Percentages and concentrations of DCs in blood

**Figure 1:**
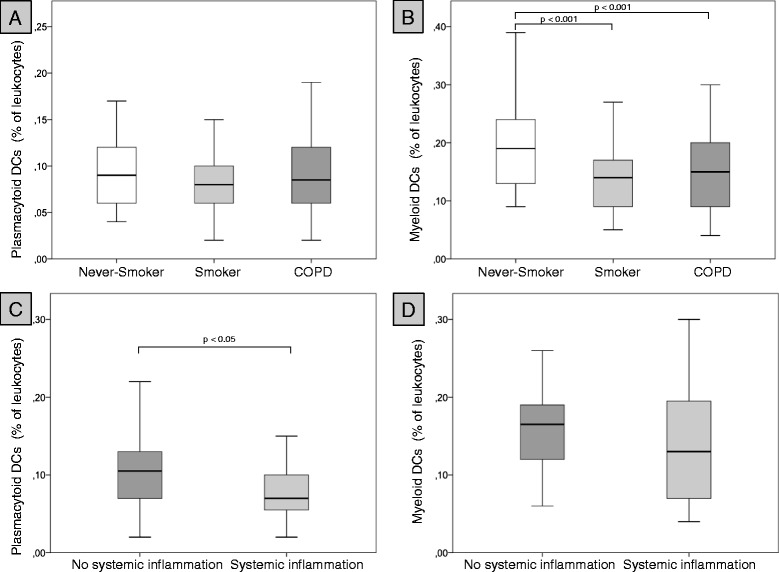
**Percentages of dendritic cells in peripheral blood.** Upper panel: percentages of plasmacytoid DCs **(A)** and myeloid DCs **(B)** among blood leukocytes of 21 never-smokers (Never-smoker), 21 smokers with normal lung function (Smoker) and 54 patients with COPD (COPD). Lower panel: percentages of plasmacytoid DCs **(C)** and myeloid DCs **(D)** in those 27 COPD patients without at least 2 elevated markers of systemic inflammation (no systemic inflammation) and those 27 COPD patients with at least 2 elevated markers of systemic inflammation (systemic inflammation).

**Figure 2:**
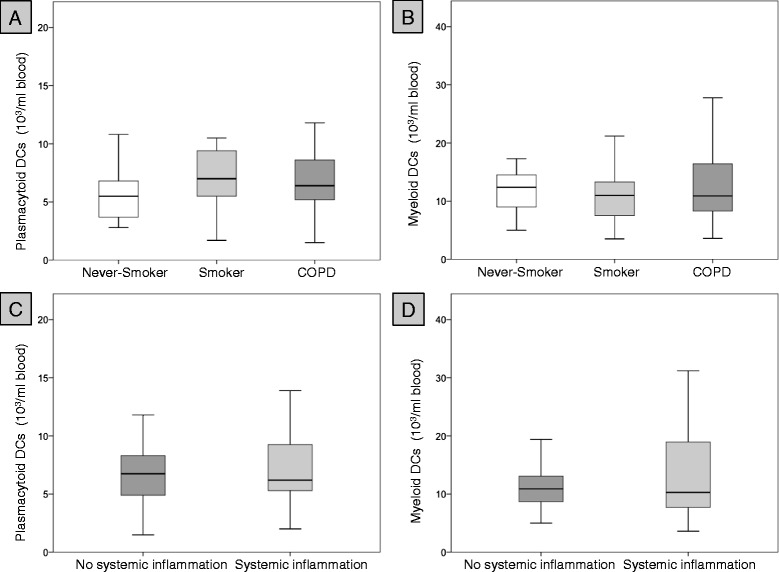
**Concentrations of dendritic cells in peripheral blood.** Upper panel: concentrations of plasmacytoid DCs **(A)** and myeloid DCs **(B)** in peripheral blood of 21 never-smokers (Never-smoker), 21 smokers with normal lung function (Smoker) and 54 patients with COPD (COPD). Lower panel: concentrations of plasmacytoid DCs **(C)** and myeloid DCs **(D)** in those 27 COPD patients without at least 2 elevated markers of systemic inflammation (no systemic inflammation) and those 27 COPD patients with at least 2 elevated markers of systemic inflammation (systemic inflammation).

Although the percentages of mDCs in blood were sigificantly higher in never-smokers than in smokers and patients with COPD, there were no differences in total mDC concentrations between the groups. Percentages and concentrations of pDCs did not differ between the groups (Table 3 and Figures 1 and 2). The ratio of mDCs to pDCs in peripheral blood did not differ between the groups (Table 3). DC concentrations in peripheral blood did not differ between former smokers (n = 42) and current smokers with COPD (n = 12) (data not shown). Percentages and concentrations of blood mDC did not differ between patients with systemic inflammation (n = 27) and patients without systemic inflammation (n = 27) (Figures 1 and 2). Although the percentage of pDCs was significantly lower in patients with systemic inflammation (n = 27), as compared with patients without systemic inflammation (n = 27), there was no difference in pDC concentrations between these two groups (Figures 1 and 2).

**Table 3:** **Dendritic cell concentrations and characteristics**

	**Never-smoker (N)**	**Smoker (S)**	**COPD (C)**	**S vs. N**	**C vs. N**	**C vs. S**
	**n = 21**	**n = 21**	**n = 54**	**p-value**	**p-value**	**p-value**
**mDC** (% of leukocytes)	0.19 [0.09-0.39]	0.14 [0.05-0.27]	0.15 [0.04-0.30]	<0.05	<0.05	0.354
**mDC** (10^3^/ml blood)	12.4 [5.0-26.5]	11.0 [3.5-29.2]	10.9 [3.6-31.2]	0.392	0.654	0.575
**pDC** (% of leukocytes)	0.09 [0.04-0.25]	0.08 [0.02-0.19]	0.09 [0.02-0.22]	0.595	0.740	0.749
**pDC** (10^3^/ml blood)	5.5 [2.8-15.4]	7.0 [1.7-19.4]	6.4 [1.5-16.9]	0.227	0.145	0.680
**ratio mDC/pDC**	1.9 [1.0-4.8]	1.3 [0.7-3.6]	1.7 [0.5-5.2]	0.084	0.141	0.575
**mDC surface markers**						
**BDCA-1** (% positive mDC)	61 [37–87]	67 [36–92]	72 [34–94]	0.505	<0.05	0.101
**BDCA-3** (% positive mDC)	41 [14–77]	68 [24–100]	61 [20–99]	<0.001	<0.001	0.516
**CD54** (MFI)	260 [177–373]	251 [87–355]	262 [160–445]	0.651	0.354	0.097
**CD86** (% positive mDC)	24 [1–70]	56 [6–77]	44 [1–92]	<0.05	<0.05	0.953
**PD-L1** (% positive mDC)	8 [0–31]	11 [1–66]	9 [0–41]	0.529	0.925	0.286
**CCR5** (% positive mDC)	9 [0–63]	12 [0–75]	28 [0–91]	0.191	<0.01	0.200
**OX40L** (% positive mDC)	9 [1–91]	12 [0–32]	16 [5–72]	0.642	<0.05	<0.01
**pDC surface markers**						
**CD54** (MFI)	316 [181–480]	351 [251–715]	287 [0–698]	0.406	0.124	<0.05
**PD-L1** (% positive pDC)	35 [4–62]	36 [9–73]	23 [0–80]	0.678	< 0.05	< 0.01

Parameters are displayed as median values [minimum…maximum]. *Abbreviations denote:* Plasmacytoid Dendritic Cell (pDC), myeloid Dendritic Cell (mDC), Blood Dendritic Cell Antigen (BDCA), Cluster of Differentiation (CD), Chemokine Receptor (CCR), Programmed Death Ligand 1 (PD-L1). The columns on the right side of the table show comparisons between the groups: Asymptomatic smokers (S), never-smokers (N), patients with COPD (C).

### Surface molecule expression on blood DCs

**Figure 3:**
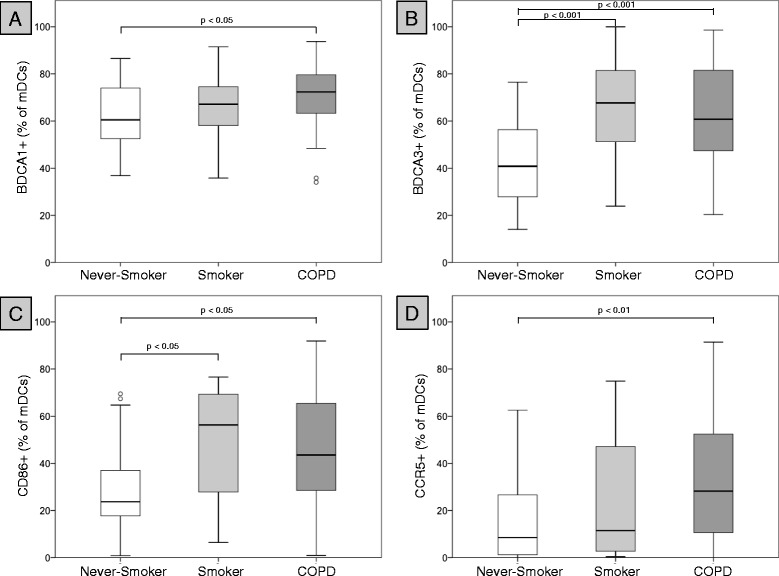
**Surface molecule expression on blood mDCs.** Boxplots show the expression (% positive blood mDCs) of BDCA-1 **(A)**, BDCA-3 **(B)**, CD86 **(C)**, CCR5 **(D)** on blood mDCs of 21 never-smokers (Never-Smoker), 21 current smokers with normal lung function (Smoker) and 54 patients with COPD (COPD).

**Figure 4:**
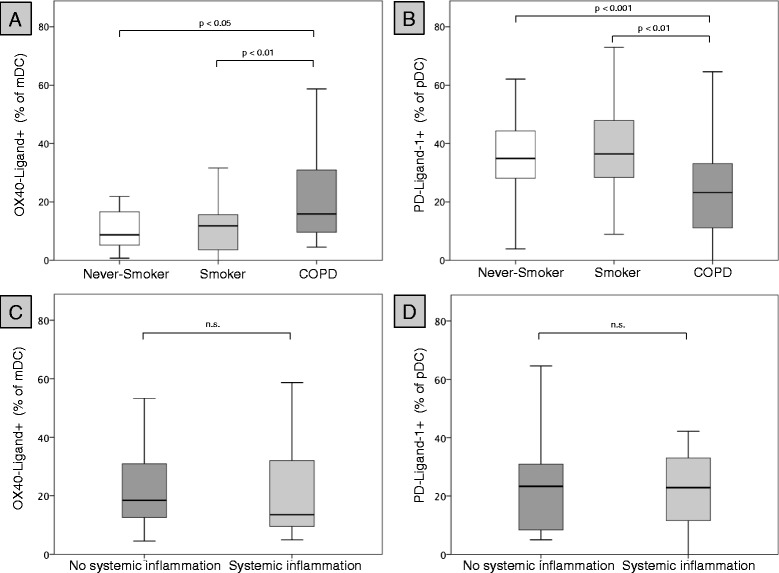
**Expression of PD-L1 and OX40L on blood DCs.** Upper panel: expression (% positive blood DCs) of OX40L on blood mDCs **(A)** and PD-L1 on blood pDCs **(B)** of 21 never-smokers (Never-Smoker), 21 current smokers with normal lung function (Smoker) and 54 patients with COPD (COPD). Lowe panel: expression (% positive blood DCs) of OX40L on blood mDCs **(C)** and PD-L1 on blood pDCs **(D)** in those 27 COPD patients with less than 2 elevated markers of systemic inflammation (no systemic inflammation) and those 27 COPD patients with at least 2 elevated markers of systemic inflammation (systemic inflammation).

**Figure 5:**
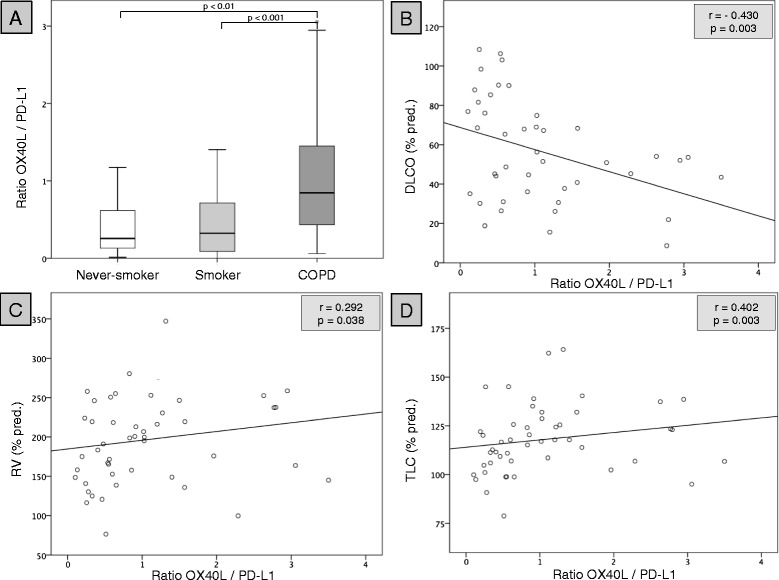
**OX40L / PD-L1 ratio and its association with emphysema.** Graph **A** shows the ratio of OX40L expression on mDCs to PD-L1 expression on pDCs (OX40L/PD-L1 ratio) of 21 never-smokers (Never-Smoker), 21 current smokers with normal lung function (Smoker) and 54 patients with COPD (COPD). The other graphs show the correlation between the OX40L/PD-L1 ratio and the diffusion capacity (DLCO) **(B)**, the residual volume (RV) **(C)** and the total lung capacity (TLC) **(D)** of the 54 patients with COPD. The Spearman’s correlation coefficient (r) and the p-value (p) is given for each correlation.

Patients with COPD displayed a significantly increased expression of BDCA-1 (Figure 3A), BDCA-3 (Figure 3B), CD86 (Figure 3C) and CCR5 (Figure 3D) on mDCs, as compared with never-smoking controls. However, the expression of these markers did not differ between asymptomatic smokers and patients with COPD (Table 3). There were no differences in the expression of CD54 and PD-L1 on mDCs between the groups (Table 3). The expression of OX40-ligand (OX40L) on mDCs was significantly higher in patients with COPD than in both control groups (Table 3 and Figure 4A). In contrast, the expression of PD-L1 on pDCs was significantly lower in patients with COPD, as compared with both control groups (Table 3 and Figure 4B). Consequently, the ratio of OX40L expression on mDCs to PD-L1 expression on pDCs (OX40L/PD-L1 ratio) was significantly increased in patients with COPD, as compared with both control groups (Figure 5A). There was no significant correlation between blood Tregs (total concentrations and percentages among CD4-positive T-cells) and the OX40L expression on mDCs, PD-L1 expression on pDCs and the OX40L/PD-L1 ratio (data not shown). CD54 expression on pDCs was significantly lower in patients with COPD than in asymptomatic smokers, but did not differ between patients with COPD and never-smoking controls (Table 3).

### Association with clinical parameters

There was no significant association of the mDC OX40L or pDC PD-L1 expression with anthropometric parameters, pulmonary function, the 6-MWT distance or the CAT score in patients with COPD (data not shown). There was no significant association of the OX40L/PD-L1 ratio with anthropometric parameters, the 6-MWT distance or the CAT score in patients with COPD (data not shown). In contrast, the OX40L/PD-L1 ratio correlated significantly with parameters of pulmonary emphysema such as the diffusion capacity (DLCO), the residual volume (RV) or the total lung capacity (TLC) (Figure 5B-D).

### Subgroup analyses

**Figure 6:**
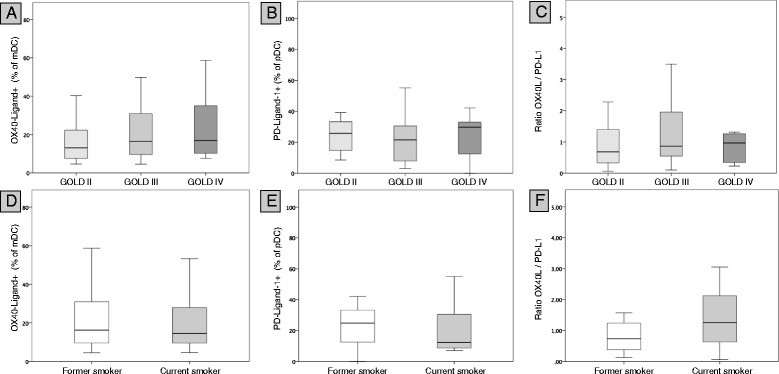
**Subgroup analyses.** The expression of OX40L on mDCs, the expression of PD-L1 on pDCs and the OX40L/PD-L1 ratio is shown for the subgroups of patients with spirometric GOLD stages II (n = 14), III (n = 26) and IV (n = 14) **(Panel A-C)** and for the subgroups of current smokers with COPD (n = 12) and former smokers with COPD (n = 42) **(Panel D-F)**. There were no significant differences between the subgroups for each parameter.

The expression of DC surface molecules did not significantly differ between current smokers (n = 12) and former smokers with COPD (n = 42)(data not shown). The expression of OX40L on mDCs, the expression of PD-L1 on pDCs and the OX40L/PD-L1 ratio did not significantly differ between patients with spirometric GOLD stages II (n = 14), III (n = 26) and IV (n = 14) (Figure 6A-C) and between current smokers (n = 12) and former smokers with COPD (n = 42)(Figure 6D-F), although the highest median OX40L/PD-L1 ratios were found in the subgroup of current smokers with COPD and in the subgroup of patients with spirometric GOLD stage IV (Figure 6C, F). Despite an increase in CCR5 expression (p = 0.044) in OCS treated patients, there were no differences in DC surface molecules expression between patients treated with OCS (n = 10) and patients not treated with OCS (n = 44). No differences in surface molecule expression was observed between patients treated with ICS (n = 31) and patients not treated with ICS (n = 23)(data not shown). Presence of systemic inflammation did not have an impact on the expression of the examined DC surface molecules. In particular, there was no significant difference in the expression of OX40L on mDCs and PD-L1 on pDCs between patients with systemic inflammation (n = 27) and patients without systemic inflammation (n = 27) (Figure 4C and D).

## Discussion

This is the first report showing an imbalance of DC co-stimulation in COPD. We demonstrate a specific increase in the pro-inflammatory co-stimulatory molecule OX40L on blood mDCs and a specific decrease in the anti-inflammatory co-stimulatory molecule PD-L1 on blood pDCs in COPD. In addition, we show that the OX40L/PD-L1 ratio, as a quantitative measure of this imbalance, correlates with the severity of pulmonary emphysema. Thus, the observed imbalance of DC co-stimulation might contribute to the pathogenesis of COPD.

Programmed death ligand 1 (PD-L1, also known as B7-H1), a member of the family of co-stimulatory B7 receptors, is expressed on DCs and binds to the CD28 family member PD-1 on T-cells [16]. The interaction of PD-L1 with PD-1 has anti-inflammatory effects by inducing regulatory T cells (Tregs) and suppressing self- and allo-reactive T cells [17-19], and plays a central role in the prevention of autoimmune and chronic inflammatory diseases [16]. PD-L1 is an important component of the anti-inflammatory functions of pDCs [20]. T cells upregulate PD-1 on the surface following activation [16]. The elevated expression of PD-1 on circulating CD4+ T cells in COPD [21] might, therefore, be related to a chronic T cell ativation in this condition. We show a decreased expression of the corresponding ligand (PD-L1) on pDCs in COPD and speculate that this defective PD-L1 expression results in a decreased ability of pDCs to control inflammation, contributing to chronic inflammation in COPD. Therefore, further studies are needed to investigate the role of the PD-L1/PD-1 axis in COPD pathogenesis.

In contrast to PD-L1, we found an increased expression of OX40L on circulating mDCs in COPD. The expression of OX40L, a costimulatory molecule of DCs binding to the OX40 receptor on T cells, has been shown to be regulated by thymic stromal lymphopoietin (TSLP) which is primarily derived from bronchial epithelial cells [22]. A TSLP-induced stimulation of the OX40L/OX40 axis has pro-inflammatory effects [22]. As a key driver of Th2 immune responses, TSLP has been postulated to play a major pathogenetic role in asthma [23]. However, an increased expression of TSLP has also been found in COPD [22,24]. In addition, there is an increased OX40 expression on circulating T cells (especially CD8+ T cells) in patients with COPD [25]. Thus, the increased OX40L expression on circulating mDCs observed in the current study suggests an upregulation of the OX40L/OX40 axis in COPD. There is an ongoing discussion on possible therapeutic implications of increased TSLP and Th2 cytokine production in COPD [26,27]. Our results suggest that the OX40L/OX40 axis might be involved in COPD pathogenesis and could represent a potential new target for COPD treatment.

In order to quantify the extent of the imbalanced expression of co-stimulatory molecules on DCs, we calculated the ratio of OX40L expression on mDCs to PD-L1 expression on pDCs (“OX40L/PD-L1 ratio”). This ratio was significantly increased in patients with COPD (as compared with both control groups) and correlated with the severity of pulmonary emphysema. These data suggest that the observed imbalance of DC co-stimulation might contribute to the complex immunopathogenesis of emphysema in COPD [28]. In contrast, we did not find a significant correlation between the OX40L/PD-L1 ratio and circulating Tregs (CD3^+^CD4^+^CD25^++^CD127^low/neg^ cells) in patients with COPD. This finding does not necessarily rule out an association between the imbalanced DC co-stimulation and altered Treg functionality in COPD as there are several methods to measure Tregs and their various subclasses and functions [21,29]. Therefore, further clinical and laboratory studies are needed to better understand the relationship between altered DC phenotypes and T cell functions in COPD.

The chemokine receptor CCR5 has been implicated in the pathogenesis of COPD [30]. CCR5 deficiency results in a marked reduction in airway inflammation and a reduced formation of peribronchial lymphoid follicles in animal models of COPD [31]. Here, we demonstrate that CCR5 is upregulated on blood mDCs in patients with COPD. CCR5 ligands such as CCL4 (MIP-1beta) and CCL5 (RANTES) are known to be increased in the airway wall of patients with COPD [32,33]. We hypothesise that an increased expression of CCR5 on blood mDCs and increased concentrations of CCR5 ligands in the airway wall lead to an selective accumulation of these mDCs in the airway wall. This accumulation has two possible implications. First, the local increase in mDCs could contribute to lymph follicle formation and small airway inflammation in COPD [1]. Second, the retention of CCR5-positive mDCs in the airway wall could result in a decrease of CCR5-positive mDCs in the airway lumen. Because CCR5 plays an important role in the uptake of microbial antigens, this decrease may contribute to an altered response to microbial antigens in COPD airways [14].

There is substantial evidence suggesting increased DC concentrations in the lung parenchyma of patients with COPD [3-6], however, there are conflicting reports on blood DC concentrations in patients with COPD [21,34]. Galgani and colleagues postulated a decrease in blood pDCs (and a resulting increase in the mDC/pDC ratio) [34], whereas Kalathil and colleagues [21] and our study did not find differences in blood pDC concentrations between patients with COPD and controls. One possible explanation for the discrepancy between the studies might be differences in the medication of the patients, the severity of the disease and the presence of different comorbidities. Therefore, further studies are needed to better understand the regulation of pDC concentrations in COPD.

It has been postulated that mDCs can be subdivided into BDCA-1-positive mDCs (“mDC1”) and BDCA-3 positive mDCs (“mDC2”) [35,36], based on a study with blood mDCs from healthy volunteers [37]. However, BDCA-3 can be upregulated on BDCA-1 positive mDCs [37]. In human airways, both BDCA-1 and BDCA-3 are expressed on > 50% of all mDCs, suggesting an overlap in the expression of both markers [10,11,14]. In the current study, both markers were upregulated on blood mDCs (expression on > 60% of mDCs) in asymptomatic smokers and in patients with COPD suggesting that the clear distinction between these two blood mDC populations is lost in these conditions. The functional consequences of these findings are currently unclear and need further investigation.

## Conclusion

This study shows, for the first time, that an imbalanced expression of co-stimulatory molecules on circulating DCs is associated with the severity of pulmonary emphysema in COPD. These findings will help to understand the immunopathogenesis of COPD.

## Additional file

Additional file 1: Table S1. Antibodies used for flow cytometry. **Figure S1.** Gating of blood dendriticc cells. **Figure S2.** Histogram plots of mDC surface markers in blood. **Figure S3.** Histogram plots of pDC surface markers in blood. **Figure S4.** Markers of systemic inflammation in peripheral blood. **Figure S5.** Correlation of clinical parameters with Interleukin-6. **Figure S6.** Regulatory T cells (Tregs) in peripheral blood.

## Abbreviations

BDCA: Blood Dendritic Cell Antigen
CCR5: C-C Chemokine Receptor 5
CD: Cluster of Differentiation
COPD: Chronic Obstructive Pulmonary Disease
DC: Dendritic Cell
FACS: Fluorescence Activated Cell Sorter
FEV_1_: Forced expiratory volume in the first second
mDC: Myeloid Dendritic Cell
OX40L: OX40 Ligand
pDC: Plasmacytoid Dendritic Cell
PD-L1: Programmed death ligand 1
TSLP: Thymic stromal lymphopoietin
