# Supplementary material for: Imbalance of dendritic cell co-stimulation in COPD
Source: Respir Res. 2015 Feb 7;16(1):19. doi: 10.1186/s12931-015-0174-x (PMC4335663; doi:10.1186/s12931-015-0174-x)
Supplement: Additional file 1: Table S1. — Antibodies used for flow cytometry. Figure S1. Gating of blood dendriticc cells. Figure S2. Histogram plots of mDC surface markers in blood. Figure S3. Histogram plots of pDC surface markers in blood. Figure S4. Markers of systemic inflammation in peripheral blood. Figure S5. Correlation of clinical parameters with Interleukin-6. Figure S6. Regulatory T cells (Tregs) in peripheral blood. [file 12931_2015_174_MOESM1_ESM.pdf]

Imbalance of dendritic cell co-stimulation in COPD

Paul Stoll, Martin Ulrich, Kai Bratke, Katharina Garbe,
J. Christian Virchow and Marek Lommatzsch

Additional File

Antibodies used for flow cytometry

Antigen	Label	Clone	Company
Lineage Cocktail 1	FITC	SK7, MfP9, 3G8, SJ25C1, L27, NCAM16.2	BD Bioscience
HLA-DR	PerCP	L243	BD Bioscience
CD11c	PE	S-HCL-3	BD Bioscience
CD11c	APC	S-HCL-3	BD Bioscience
CD123	PE	9F5	BD Bioscience
CD123	APC	AC145	Miltenyi Biotec
BDCA1	APC	AD5-8E7	Miltenyi Biotec
BDCA3	APC	AD5-14H12	Miltenyi Biotec
CD54	APC	HA58	BD Bioscience
CD86	APC	BU63	Life Technologies
CCR5	APC	2D7	BD Bioscience
OX40L	PE	11C3.1	BioLegend
PD-L1	APC	29E.2A3	BioLegend
CD3	PerCP	SK7	BD Bioscience
CD4	FITC	SK3	BD Bioscience
CD25	PE	ACT-1	DAKO
CD127	APC	A019D5	BioLegend

Table S1. Antibodies used for flow cytometry
Abbreviations denote: APC, allophycocyanin; BDCA, blood dendritic cell antigen; CD, cluster of differentiation; FITC, fluorescein isothiocyanate; PE, phycoerythrin; PerCP, peridinin chlorophyll protein. *Manufacturers:* BD Bioscience, Heidelberg, Germany; BioLegend, Fell, Germany; DAKO, Hamburg, Germany; Life Technologies, Darmstadt, Germany;
Miltenyi Biotec, Bergisch Gladbach, Germany.

Gating strategy

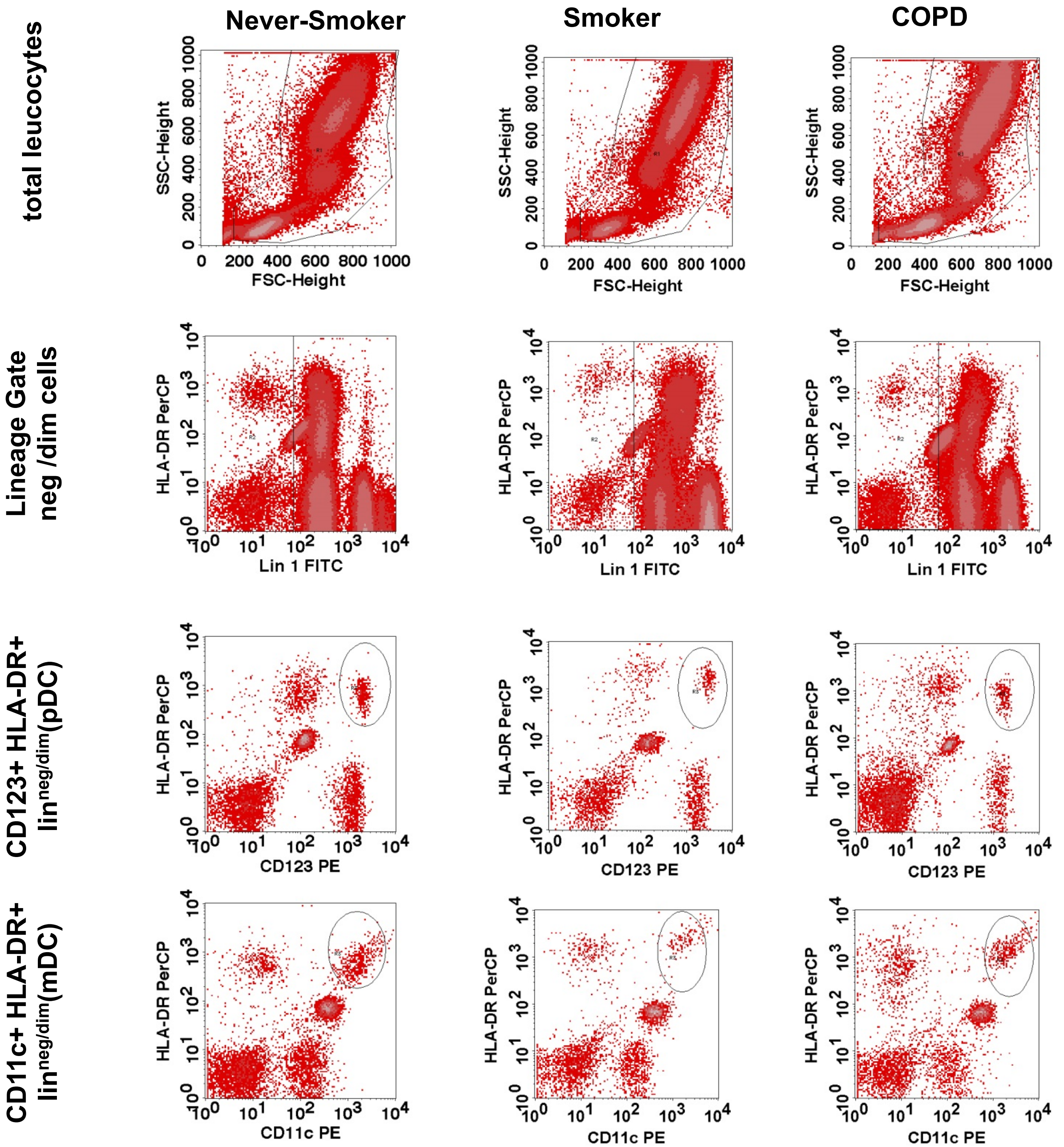

Figure S1. Gating of blood dendritic cells

Total cells were gated in a FSC/SSC-plot (first row). In this cell population, lineage negative/dim (lin $^{neg/dim}$) cells were gated (second row). In the lin $^{neg/dim}$ gate, cells expressing HLA-DR and CD11c were identified as mDCs (linneg/dimHLA-DR+CD123+)(third row), and cells expressing HLA-DR and CD123 were identified as pDCs (linneg/dimHLA-DR+CD11c+)(fourth row). The figure shows the blood cells of a never-smoker (first column), a smoker with normal lung function (second column) and a patient with COPD (third column).

Histogramm Plots mDCs (Part 1)

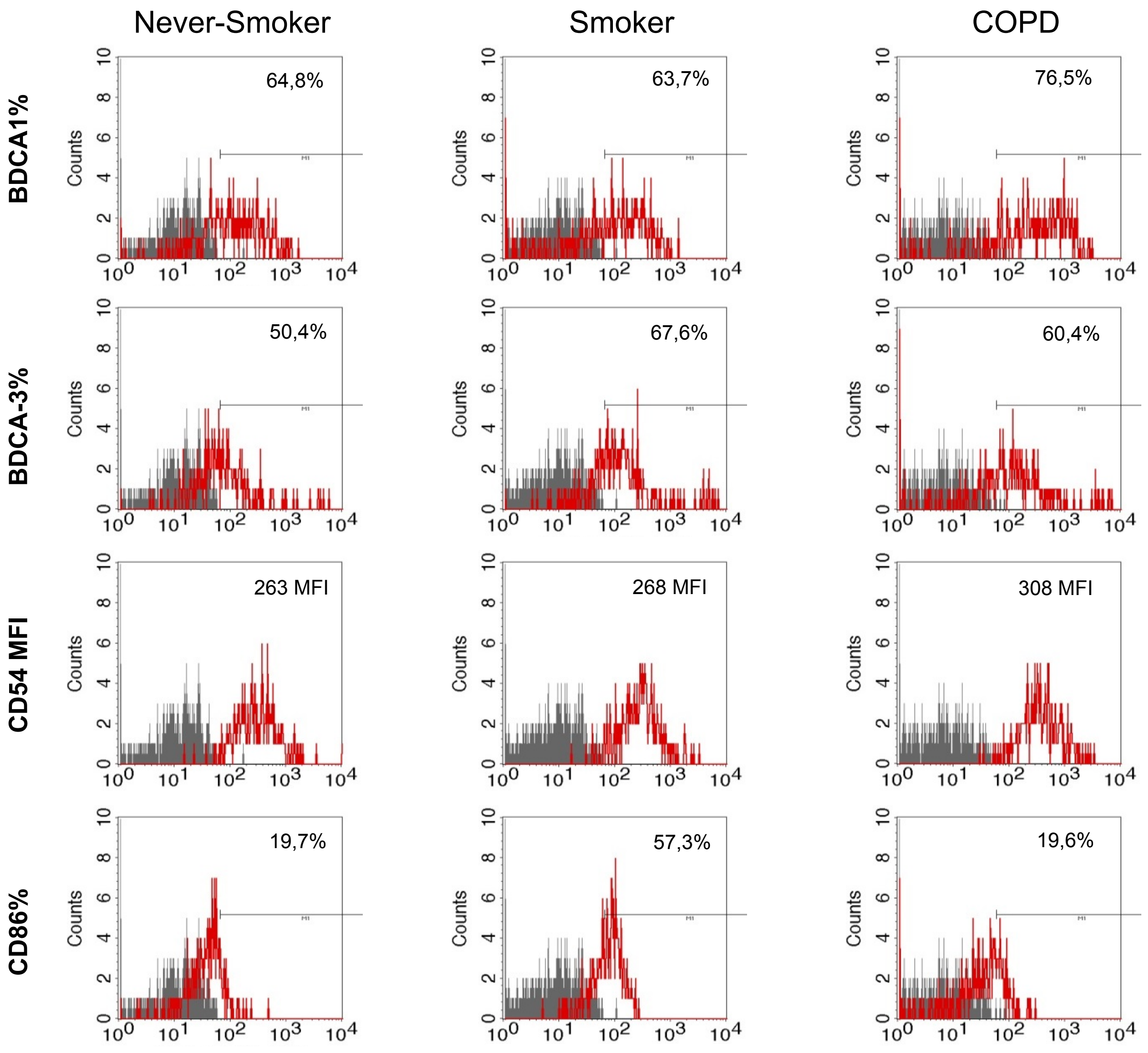

Histogramm Plots mDCs (Part 2)

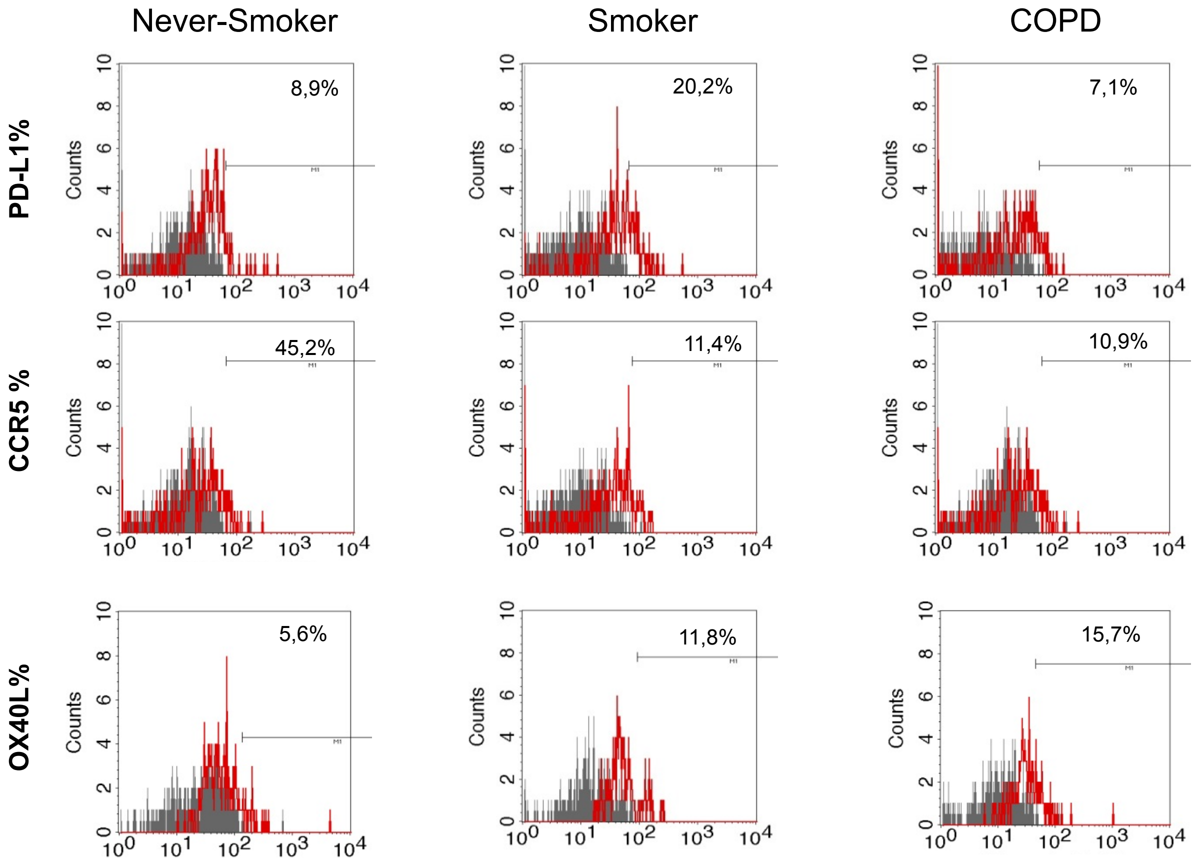

Figure S2. Histogram plots of mDC surface markers in blood
Myeloid DCs were identified as lin $^{neg/dim}$ HLA-DR+CD11c+ cells. Histograms show the staining of these cells with antibodies against surface markers (marker antibody, red) compared to the staining with respective isotype control antibodies (control antibody, grey). The figure shows examples from a never-smoker (first column), a smoker with normal lung function (second column) and a patient with COPD (third column).

Histogramm Plots pDCs

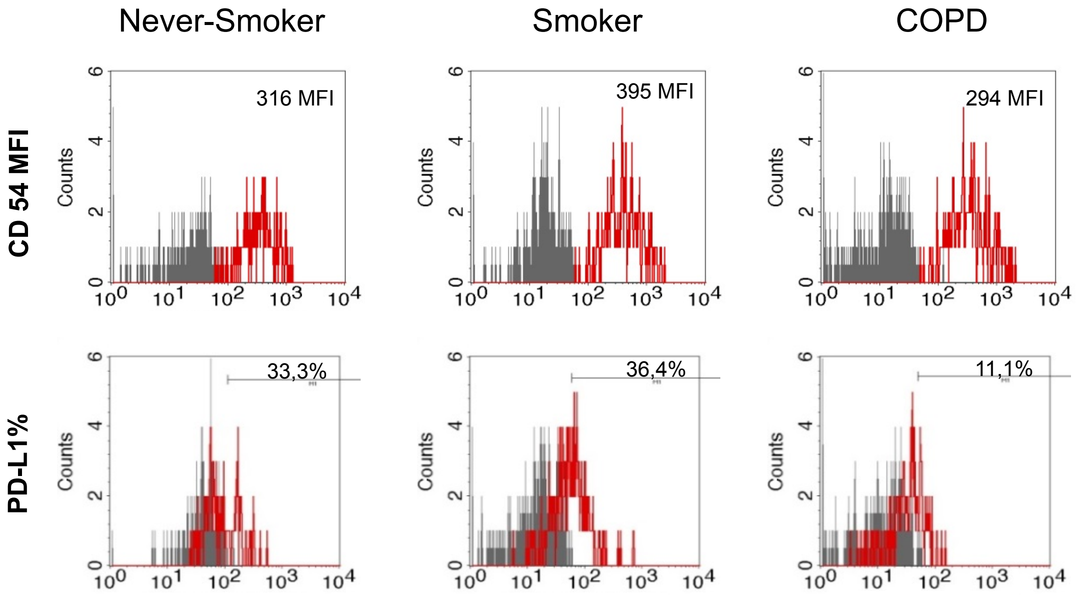

Figure S3. Histogram plots of pDC surface markers in blood
Plasmacytoid DCs were identified as lin $^{neg/dim}$ HLA-DR+CD123+ cells. Histograms show the staining of these cells with antibodies against surface markers (marker antibody, red) compared to the staining with respective isotype control antibodies (control antibody, grey). The figure shows examples from a never-smoker (first column), a smoker with normal lung function (second column) and a patient with COPD (third column).

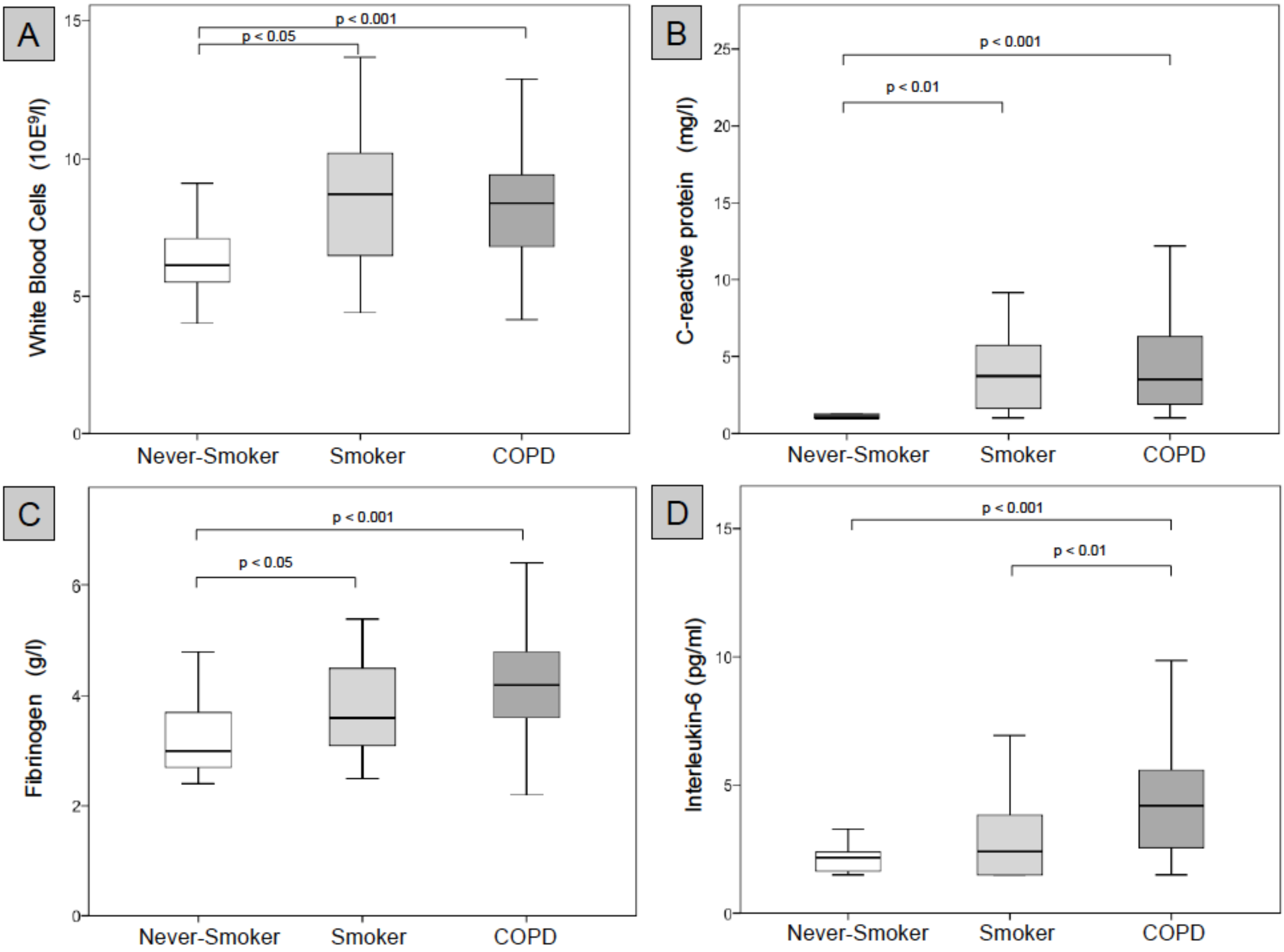

Figure S4. Markers of systemic inflammation in peripheral blood
Shown are concentrations of leukocytes (A), C-reactive protein (B), Fibrinogen (C) and Interleukin-6 (D) of 21 never-smokers (Never-smoker), 21 smokers with normal lung function (Smoker) and 54 patients with COPD (COPD).

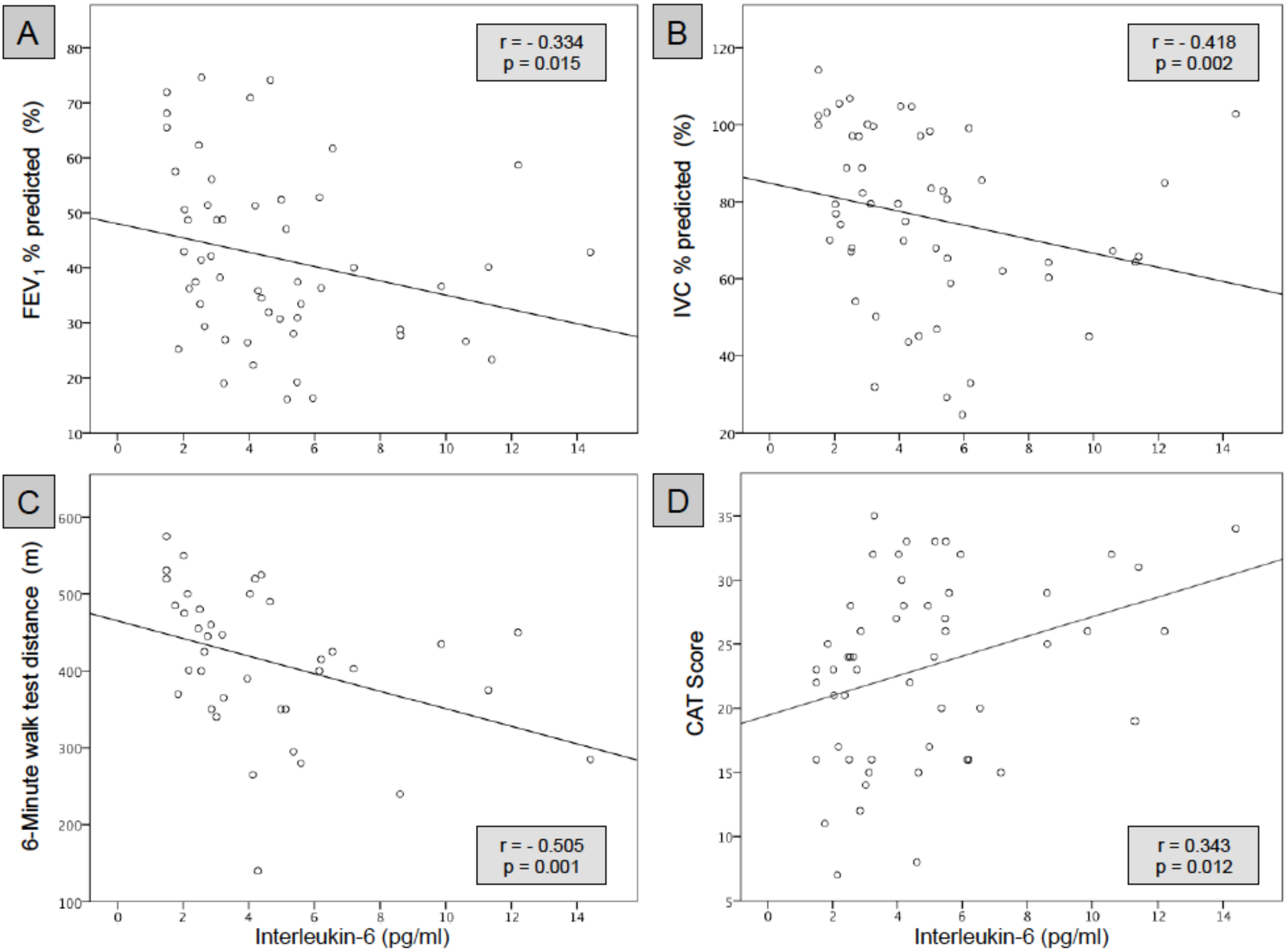

Figure S5. Correlation of clinical parameters with Interleukin-6

Shown are correlations of Interleukin-6 concentrations in peripheral blood with the forced expiratory capacity (FEV1) (A), the inspiratory vital capacity (IVC) (B), the 6-Minute walk test distance (C) and the COPD assessment test (CAT) score (D) of 54 patients with COPD.
The Spearman´s correlation coefficient (r) and the p-value (p) is shown for each correlation.

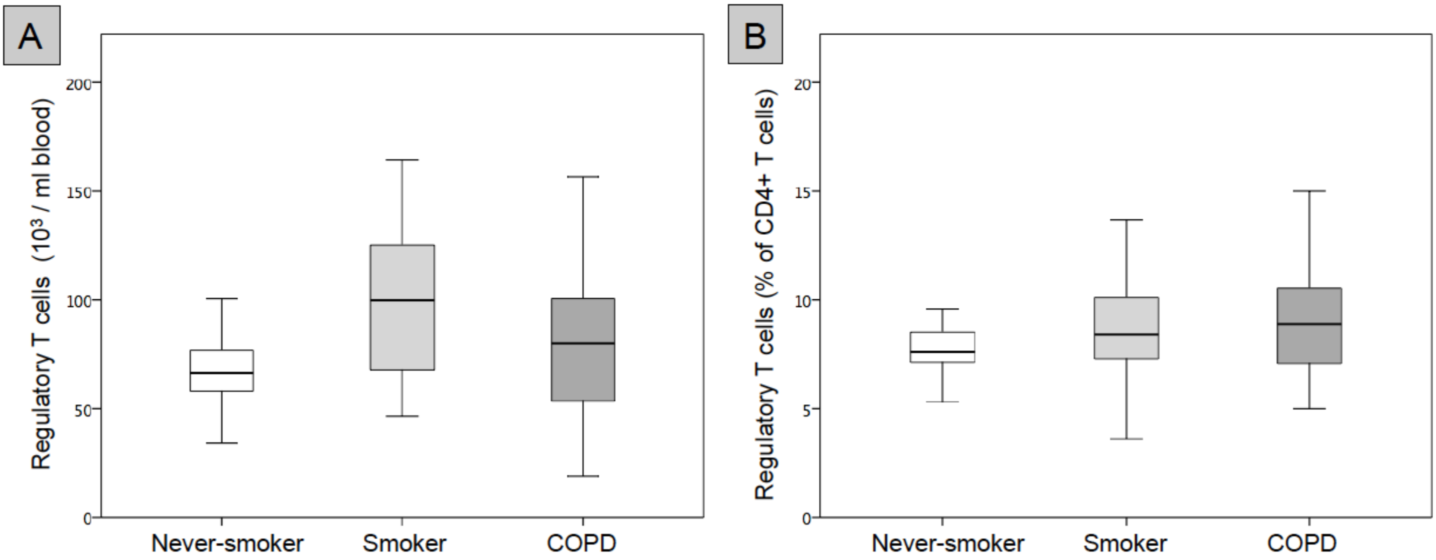

Figure S6. Regulatory T cells (Tregs) in peripheral blood
Shown are total concentrations of Tregs (A) and the percentage of Tregs among CD4+ T cells (B) in peripheral blood of 21 never-smokers (Never-smoker), 21 smokers with normal lung function (Smoker) and 54 patients with COPD (COPD). There were no significant differences between the groups.
